# Tri-Layered Full-Thickness Artificial Skin Incorporating Adipose-Derived Stromal Vascular Fraction Cells, Keratinocytes, and a Basement Membrane

**DOI:** 10.3390/bioengineering12070757

**Published:** 2025-07-12

**Authors:** Jung Huh, Seong-Ho Jeong, Eun-Sang Dhong, Seung-Kyu Han, Kyung-Chul Moon

**Affiliations:** 1 Korea University College of Medicine, Seoul 02841, Republic of Korea; 2 Department of Plastic Surgery, Korea University College of Medicine, Seoul 02841, Republic of Korea

**Keywords:** artificial skin, basement membrane, keratinocyte, stromal vascular fraction, wound healing

## Abstract

Tissue-engineered artificial skin has the potential to enhance wound healing without necessitating extensive surgical procedures or causing donor-site morbidity. The purpose of this study was to examine the possibility of developing tri-layered tissue-engineered full-thickness artificial skin with a basement membrane for clinical use to accelerate wound healing. We engineered full-thickness artificial skin with a basement membrane for wound healing by employing stromal vascular fraction (SVF) cells for the dermal layer and autologous keratinocytes for the epidermal layer. The fabrication of a basement membrane involved the use of 100% bovine collagen and 4% elastin produced through a low-temperature three-dimensional printer. Scaffolds for cells were printed with 100% bovine collagen. The basement membrane underwent evaluations for collagenase degradation, tensile strength, and structural characteristics using scanning electron microscopy. The final tri-layered full-thickness artificial skin included two cell scaffolds with a basement membrane between them. The basement membrane may support cellular attachment without inducing significant cytotoxic effects. This study presents a novel strategy for full-thickness artificial skin development, combining SVF and keratinocytes with an optimized collagen-elastin basement membrane. This method may overcome the significant limitations of current artificial skin, thereby contributing to the advancement of tissue-engineering in wound healing for clinical use.

## 1. Introduction

The skin serves vital functions, including protection against pathogens, regulation of body temperature, and prevention of fluid loss. Severe skin injuries, such as acute or chronic wounds, can significantly impair these essential roles, leading to increased risks of infection, osteomyelitis, dehydration, and metabolic complications [1]. Although autologous skin grafts and flap reconstruction are regarded as the standard treatments for severe skin injuries, their use is often restricted due to the availability of donor sites and the associated morbidity. These limitations underscore the urgent need for alternative wound management strategies. Therefore, interest in skin substitutes has grown and may offer promising solutions for the effective coverage of large wounds [2].

Recent advancements in wound healing technology have resulted in the creation of various artificial dermal substitutes [3,4]. However, these substitutes often lead to noticeable and undesirable scarring [5,6]. A primary challenge is wound contraction, which plays a crucial role in minimizing scar formation following complete healing. Although artificial dermis can be utilized for wound coverage, the lack of cellular components typically results in delayed healing and abnormal scar formation, including scar contractures. Recent developments in wound healing technologies have enabled the implementation of cell-based therapies and tissue-engineered artificial skin as an alternative to traditional treatments and a way to overcome the limitations associated with artificial dermis [7].

Tissue-engineered artificial skin has the capability to improve wound healing while minimizing the need for extensive surgical interventions and reducing the risk of donor site complications. This innovative approach is applicable to both acute and chronic wounds and represents a promising strategy for improving tissue regeneration [8]. Cryopreserved human fibroblast-derived dermal substitutes, such as Dermagraft (Shire Regenerative Medicine) [9] and Apligraf (Organogenesis) [10], are allogeneic skin replacements that have been shown to be effective for wound healing. Additionally, non-cryopreserved fresh human fibroblast allografts have demonstrated safety and effectiveness as treatment options for these ulcers. A bioengineered dermal substitute known as Hyalograft 3D, which consists of cultured autologous fibroblasts seeded on a hyaluronic acid scaffold, has also been developed.

The current artificial skin substitutes and tissue engineering strategies encounter several challenges that limit their clinical effectiveness. Traditional methods typically utilize multi-layered structures to replicate the architecture of natural skin, which necessitates complex fabrication processes and the careful management of individual dermal and epidermal components [11]. Although recent advancements in three-dimensional (3D) bioprinting have shown promise in developing sophisticated skin constructs with precise cell placement and biomaterial distribution [12], these techniques often require considerable technical expertise, specialized equipment, and significant financial investment [13].

Cell scaffolds feature precisely controlled density variations within a unified structure, enabling the spatial organization of keratinocytes and stromal vascular fraction (SVF) cells. This study utilized 3D printing to fabricate a basement membrane to mimic the natural papillary epidermal–dermal skin junction. This structure was strategically placed between the epidermal and dermal layers to maintain cellular separation and prevent unintended mixing.

The purpose of this in vitro study was to investigate the feasibility of developing tri-layered tissue-engineered full-thickness artificial skin, incorporating a basement membrane for clinical applications aimed at accelerating wound healing. Our objectives included establishing a rapid fabrication protocol that maintains cell functionality, achieving a structural organization that closely resembles native skin architecture, and assessing mechanical stability and biocompatibility. This innovative approach may represent a significant advancement over existing substitutes by integrating the regenerative potential of SVF cells with the epidermal function of keratinocytes within a biomimetic structure designed for the wound healing of full-thickness skin and soft tissue defects.

## 2. Materials and Methods

This study protocol was approved by the Institutional Review Board and written informed consent was obtained from the patient prior to surgery. This study was conducted in full accordance with the principles of the Declaration of Helsinki.

### 2.1. Preparation of Stromal Vascular Fraction Cells for the Dermal Layer

**Figure 1:**
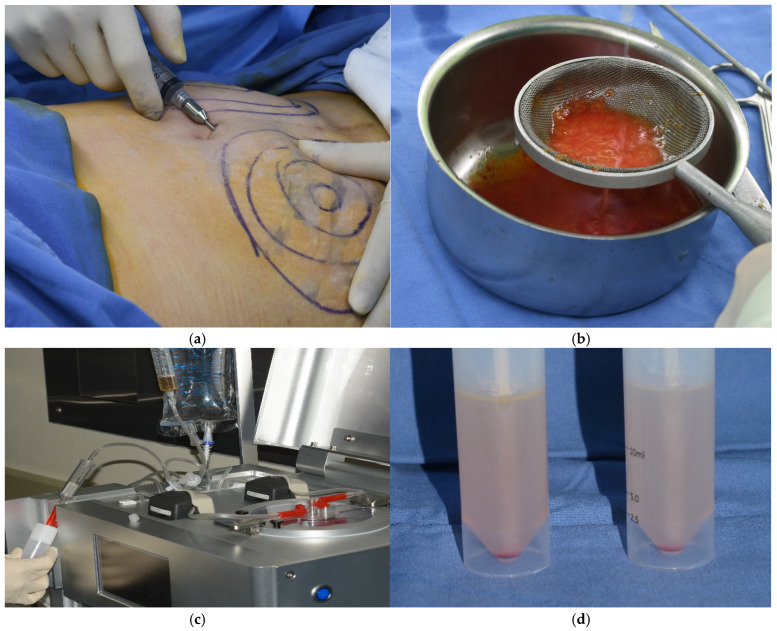
Isolation of stromal vascular fraction (SVF) cells. (**a**) Liposuction. (**b**) Washing adipose tissues. (**c**) Automated SVF cell isolation system. (**d**) SVF cells. The SVF cells were obtained from abdominal adipose tissue using an automated cell isolation system within 50 min.

Abdominal adipose tissue was obtained from a patient by liposuction. A small incision was made in the umbilical area, and a local anesthetic solution was administered using a blunt Lamis infiltration cannula. The adipose tissue was aspirated using a 50 mL syringe connected to a cannula with a 3 mm inner diameter designed to maintain the integrity of the adipose tissue. The plunger of the syringe was retracted gently during the aspiration process to avoid creating excessive negative pressure that could lead to tissue rupture. The collected adipose tissue was then processed to isolate autologous SVF cells utilizing an automated cell isolation system (Cellunit; CGBio Inc., Seoul, Republic of Korea). This apparatus features a device along with sterile, disposable cartridges tailored for the processes of tissue digestion, washing, and waste management. In brief, the harvested samples underwent enzymatic digestion with 0.1% collagenase (SERVA Electrophoresis GmbH, Heidelberg, Germany) inside a processing chamber. The digested tissue was subsequently washed with saline to eliminate any leftover collagenase before centrifugation to separate the SVF cells, which were harvested within 50 min (Figure 1).

### 2.2. Preparation of Keratinocytes for the Epidermal Layer

**Figure 2:**
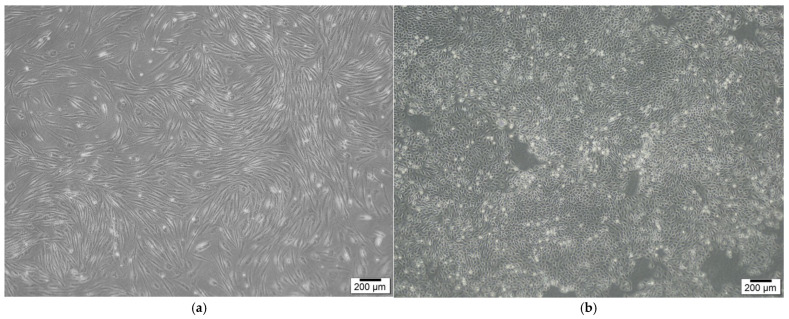
SVF cells and keratinocytes for dermal and epidermal layers. (**a**) SVF cells. (**b**) Keratinocytes. SVF cells and keratinocytes were isolated from adipose and skin tissues, respectively.

Keratinocytes were sourced from a skin biopsy and cultured in Dulbecco’s Modified Eagle Medium (DMEM; Gibco), enriched with 10% fetal bovine serum (FBS; Gibco) and 1% penicillin-streptomycin (Gibco). The cultures were kept at 37 °C in a humidified environment with 5% CO_2_. Once the adherent monolayer achieved 70–80% confluence, the cells were detached using TrypLE Express (Gibco), which contains 0.25% trypsin. After detachment, the cells were washed, re-suspended in DMEM supplemented with 10% FBS, and sub-cultured. The cells were then incubated for 24 h (Figure 2).

### 2.3. Cell Layer Scaffolds

Cell scaffolds were fabricated using 100% bovine collagen in a low-temperature 3D printer. Three-dimensional printing created a single-layered structure, which was chemically crosslinked for mechanical stability and freeze-dried to develop a multiporous architecture for cell fixation. This design streamlines the manufacturing process and clinical application by minimizing handling challenges and enhancing the scaffold’s adaptability to various wound environments.

### 2.4. Basement Membrane Between the Cell Layer Scaffolds

A basement membrane for use between the scaffolds in the full-thickness artificial skin was fabricated using 100% bovine collagen and elastin in a low-temperature 3D printer. Scaffolds with collagen-to-elastin ratios of 100:4 were printed. Three-dimensional printing created a dense thin film structure, which was chemically cross-linked for mechanical stability and freeze-dried to develop a stable architecture for cell–cell interaction without mixing the two cell layers.

### 2.5. Analysis of the Basement Membrane and Tri-Layered Full-Thickness Artificial Skin

Degradation and tensile strength tests were performed to analyze the basement membrane composed of collagen and elastin. Biocompatibility was also assessed by seeding SVF cells and keratinocytes onto the basement membrane and evaluating cell viability and cytotoxicity over a 7-day period. Keratinocytes and SVF cells were seeded onto a UV-sterilized 1 × 1 cm collagen-elastin based basement membrane and cultured under standard conditions (37 °C, 5% CO_2_) to assess the biocompatibility of the fabricated full-thickness artificial skin constructs. Cytotoxicity and cell viability were evaluated using the Cell Counting Kit-8 (CCK-8; Sigma-Aldrich, St. Louis, MO, USA) assay, and absorbance was measured at 450 nm. The CCK-8 assay was conducted at 1, 3, 5, and 7 days of culture to assess cell viability and cytotoxic effects. A commercial acellular artificial dermis made entirely of collagen sourced from porcine skin (Insuregraf, Atozbio, Seoul, Republic of Korea) was employed as the control.

**Figure 3:**
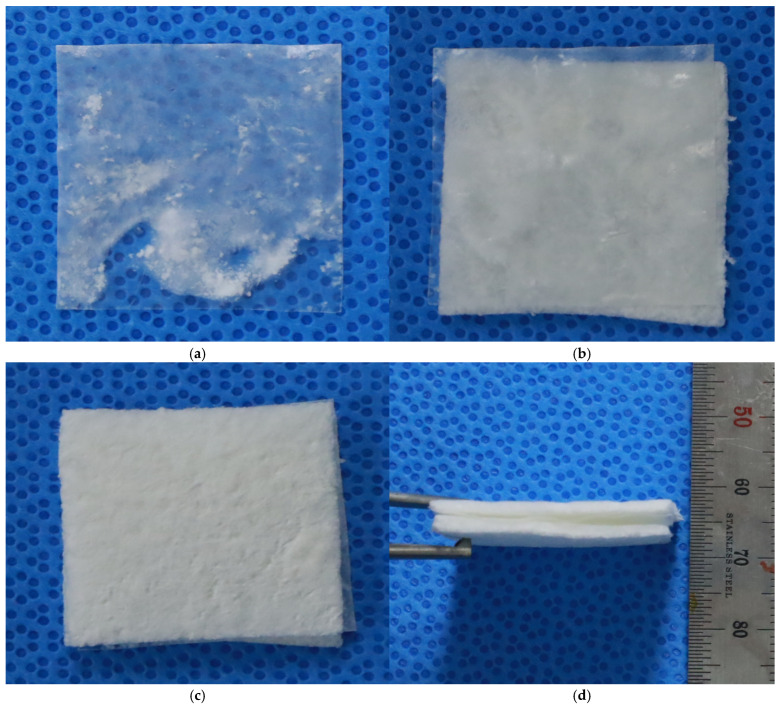
Tri-layered full-thickness artificial dermis. (**a**) Basement membrane. (**b**) Basement membrane and a cell scaffold. (**c**,**d**) Tri-layered full-thickness artificial dermis. The final tri-layered full-thickness artificial skin included SVF cell and keratinocyte scaffolds with a basement membrane between the two cell scaffolds.

The final tri-layered full-thickness artificial skin included two cell scaffolds with a basement membrane between them (Figure 3). Scanning electron microscopy (SEM) was utilized to examine the structural characteristics of the basement membrane and the final structural features of the tri-layered full-thickness artificial skin.

### 2.6. Cell Imaging of the Tri-Layered Full-Thickness Artificial Skin

The cell membranes of keratinocytes and SVF cells were labeled by 2μM PKH26 red fluorescent dye and green fluorescent dye (Sigma-Aldrich), respectively. Subsequently, cells were cultured on collagen scaffolds to create the full-thickness artificial skin. Keratinocytes were applied to the epidermal layer, while SVF cells were applied to the dermal layer, with a basement membrane laminated between each layer to fabricate the complete construct. The fabricated full-thickness artificial skin was placed on a cell culture insert (Millicell, Millipore, Burlington, MO, USA) and cultured for 5 days under standard conditions (37 °C, 5% CO_2_). For imaging purposes, the cultured artificial skin was washed three times with phosphate-buffered saline (PBS) and fixed in 4% paraformaldehyde for 12 h. After fixation, the samples were rinsed three times with PBS, agarose-embedded, and sectioned to a thickness of 30 μm using a vibratome (Leica VT1000S). The sectioned samples were mounted on slides, and fluorescence expression was captured using an EVOS FL2 fluorescence microscope (Thermo Fisher Scientific, Waltham, MO, USA).

## 3. Results

### 3.1. Analysis of the Basement Membrane

**Figure 4:**
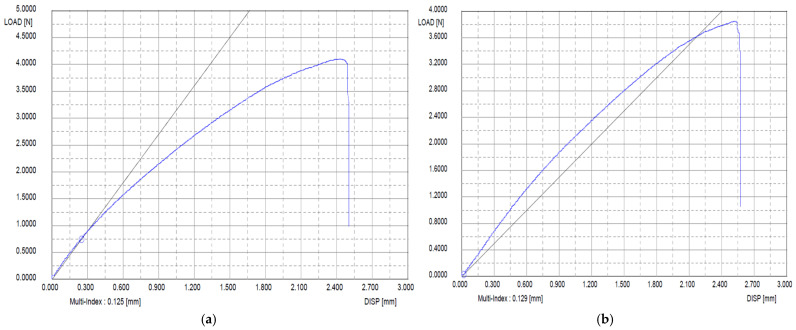
Tensile strength of the basement membrane before and after gamma sterilization. (**a**) Before gamma sterilization. (**b**) After gamma sterilization. After gamma sterilization, the basement membrane exhibited a slight reduction but maintained strength. Blue Line = Tensile strength curve of the basement memebraine; Black Line = A linear reference line.

The degradation stability of the basement membrane and acellular artificial dermis was tested under 200 U collagenase treatment. By 24 h, the commercial acellular artificial dermis was completely degraded, while the basement membrane retained the highest structural integrity, indicating its stability. The basement membrane was reduced by half by 3 days and fully degraded by 7 days. Tensile strength tests were conducted on the scaffolds with an acellular artificial dermis and basement membrane. The acellular artificial dermis showed 0.8 N and 0.3 N in the dry and wet tensile strength tests, respectively. The basement membrane showed a tensile strength of 4.2 N. After gamma sterilization, it exhibited a slight reduction from 4.2 N to 3.8 N, maintaining superior strength (Figure 4). Tensile strength was also assessed under preparation conditions of primary freeze-drying, 1-ethyl-3-dimethylaminopropyl crosslinking, washing, and secondary freeze-drying. The basement membrane also revealed superior tensile strength performance in all of these conditions compared to the acellular artificial dermis.

### 3.2. Biocompatibility

**Figure 5:**
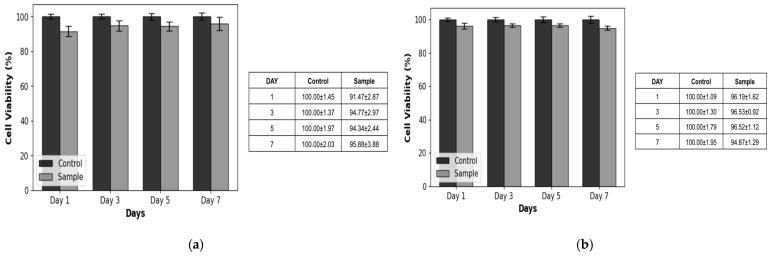
Cell viability test results. (**a**) Keratinocytes. (**b**) SVF cells. The results revealed that the fabricated basement membrane was minimally toxic to both keratinocytes and SVF cells over the 7-day culture period.

The biocompatibility results revealed that the fabricated basement membrane was minimally toxic to both keratinocytes and SVF cells during the 7-day culture period. Cell viability, expressed as a percentage of the control group, demonstrated the consistent growth and proliferation of both cell types. Keratinocyte viability tests displayed an initial viability of 91.47 ± 2.87% on day 1, which gradually increased to 95.88 ± 3.88% by day 7. Similarly, the viability of SVF cells was 96.19 ± 1.62% on day 1, which slightly improved to 94.87 ± 1.29% on day 7. These results confirmed that the basement membrane could support cellular attachment and proliferation without inducing significant cytotoxic effects (Figure 5).

### 3.3. Structural Features of the Full-Thickness Artificial Skin with a Basement Membrane

The surface morphology and structural features of the basement membrane and full-thickness artificial skin with a basement membrane were analyzed using SEM to evaluate the effects of elastin incorporation, density gradients, and tri-layer configurations. These analyses aimed to assess uniformity, pore size, and surface characteristics critical for mechanical stability.

**Figure 6:**
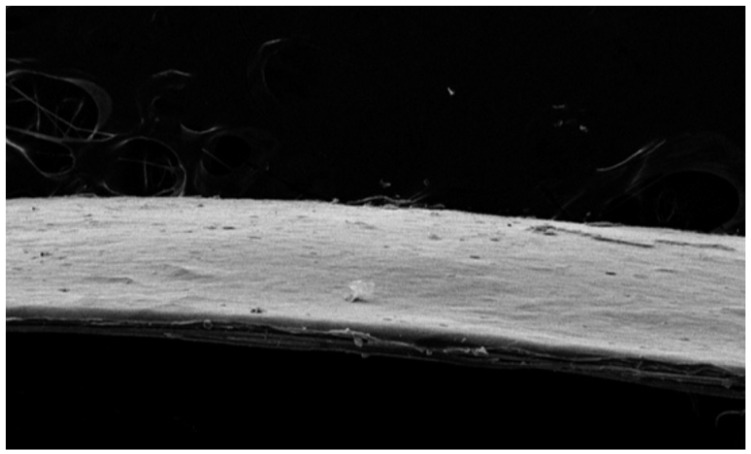
Scanning electron microscopy (SEM) image of the collagen-elastin basement membrane. The basement membrane containing 4% elastin demonstrated uniform pore sizes and smooth surface patterns, suggesting enhanced structural consistency and mechanical stability.

**Figure 7:**
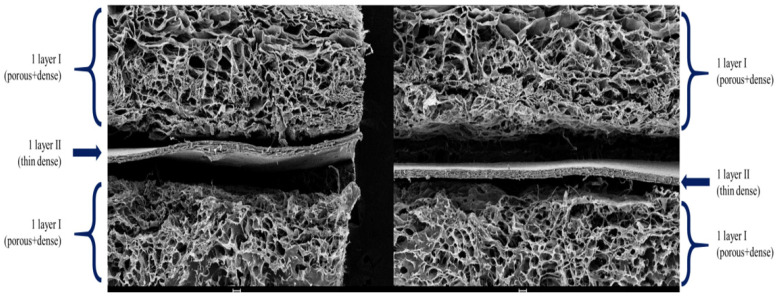
SEM image of tri-layered full-thickness artificial skin. This hierarchical structure replicates the natural dermal-epidermal interface.

The SEM images revealed significant differences between scaffolds composed of collagen for the cell layers and a collagen-elastin basement membrane. The porous regions displayed pore sizes between 100 and 300 μm, whereas the dense regions were characterized by a lack of visible pores, resulting in a smooth and uniform basement membrane. The collagen cell scaffolds exhibited large pore sizes for cell attachment, with a visible directional texture. In contrast, the basement membrane constructs containing 4% elastin demonstrated smaller but more uniform pore sizes and smoother surface patterns, suggesting enhanced structural consistency and mechanical stability compared to collagen cell scaffolds. This hierarchical structure replicates the natural dermal-epidermal interface, providing both functional and structural advantages (Figure 6 and Figure 7).

**Figure 8:**
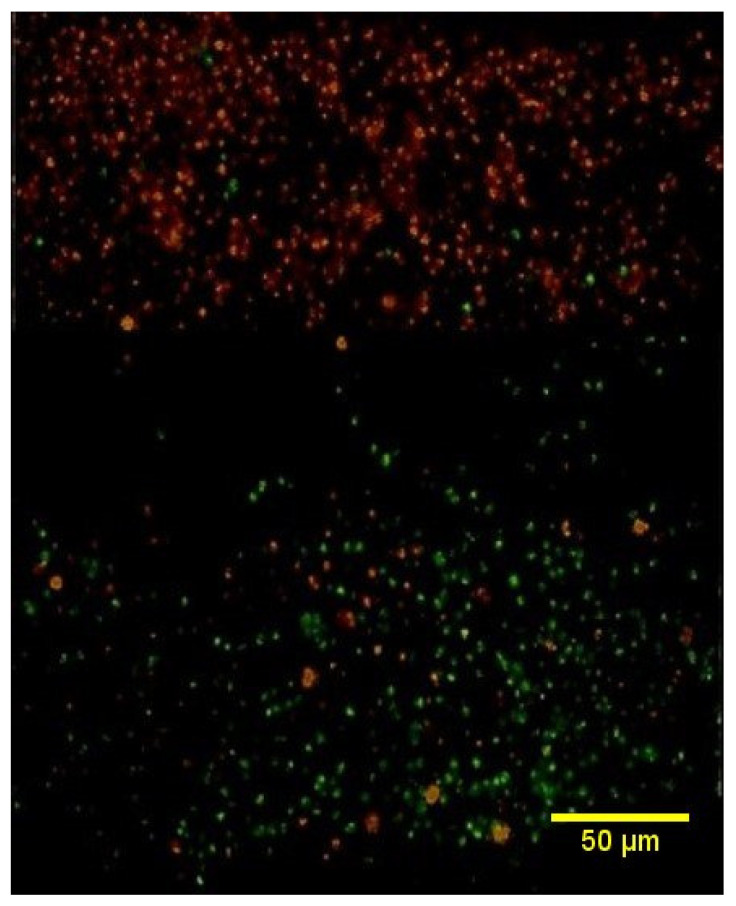
Cell image of the tri-layered full-thickness artificial skin. This image displayed red-stained keratinocytes representing the epidermal layer and green-stained SVF cells corresponding to the dermal layer, with a basement membrane positioned between the two layers.

Cell imaging results confirmed the presence of red-stained keratinocytes in the epidermal layer and green-stained SVF cells in the dermal layer, with a basement membrane placed between the two layers. This configuration facilitated potential cell-to-cell interactions (Figure 8).

## 4. Discussion

Full-thickness skin and soft tissue defects, including acute and chronic wounds, represent a significant global healthcare burden, with extensive full-thickness injuries often exceeding the natural healing capacity. While autologous skin grafting and flap reconstruction remain the gold standard, their application is limited by donor site availability and associated morbidity [14]. Recent advances in tissue engineering have led to various artificial skin substitutes, from collagen scaffolds to bioprinted constructs, typically involving biomaterial scaffolds seeded with cultured autologous keratinocytes or fibroblasts [15,16].

However, keratinocytes are epithelializing cells in superficial wounds and fibroblasts are involved in granulation tissue growth in deep and chronic wounds. Because each cell has a different effect on wound healing, the wounds to which each can be applied are limited. These approaches also face significant limitations, including extended fabrication times due to cell expansion requirements and challenges in achieving adequate host tissue integration. Furthermore, the existing substitutes often fail to fully replicate the complex structural and functional characteristics of native skin tissue [17]. Therefore, full-thickness artificial skin, composed of epidermal and dermal layers, is necessary for better wound healing in full-thickness skin and soft tissue defects.

SVF cells can be obtained in one hour and present a promising alternative, offering rapid extraction and a heterogeneous population of regenerative cells, including fibroblasts, stromal cells, endothelial progenitors, and immune cells [18]. This diverse cellular composition potentially facilitates better tissue integration and vascularization compared to purified cell populations. When combined with autologous keratinocytes, which can be efficiently isolated and expanded from small skin biopsies [19], this approach enables the construction of immunologically compatible skin substitutes. Despite these advantages, there remains a significant gap in developing clinically viable full-thickness artificial skin models that effectively mimic native tissue architecture while maintaining practical feasibility [20].

This study successfully developed a biomimetic full-thickness artificial skin construct by integrating SVF and autologous keratinocytes onto a tri-layered collagen-elastin scaffold. The incorporation of elastin enhanced mechanical properties, nearly doubling the tensile strength and improving resistance to enzymatic degradation compared to collagen-only scaffolds and acellular artificial dermis. Importantly, the construct retained substantial mechanical integrity (3.9 N) after gamma sterilization, indicating its robustness for clinical applications.

The tri-layered architecture, characterized by SEM analysis, effectively replicates the natural dermal-epidermal interface. Uniform pore distribution (100–300 μm) in the porous layers facilitates nutrient diffusion and cellular infiltration, while the dense barrier basement membrane provides structural separation and mechanical support. This hierarchical design addresses a key limitation of the existing full-thickness artificial skin substitutes, which often fail to mimic the complexity of native skin tissue [17].

The excellent biocompatibility observed in keratinocytes and SVF cells over 7 days demonstrated the suitability of the basement membrane as a septum to which both cells can adhere. The rapid isolation of SVF cells and diverse cellular composition, including endothelial progenitors, offers significant advantages over traditional fibroblast-based methods, potentially enhancing vascularization and integration [18]. These attributes address critical challenges in tissue engineering, particularly the need for rapid fabrication and effective host-tissue integration. Long-term culture observations further validated the biocompatibility of the constructs. Both keratinocytes and SVF cells showed consistent growth trends, with no signs of cytotoxicity or abnormal cellular morphology throughout the 7-day culture period. The CCK-8 assay results aligned with the microscopic findings, indicating that the fabricated constructs provide a favorable microenvironment for cellular growth and function.

The present study had some limitations. First, due to the lack of direct comparative data between SVF cells and fibroblasts, the extent to which SVF cells contribute to granulation tissue formation relative to fibroblasts remains unclear. Second, additional biomaterials, such as fibrin glue, capable of handling tri-layered full-thickness artificial skin without significant cell loss are needed. The CCK-8 assay alone may not provide a comprehensive evaluation of cytotoxicity and cell proliferation. Incorporating additional methods, such as immunostaining and flow cytometry to assess cell apoptosis, would strengthen claims of biocompatibility. Additionally, variability in SVF composition between donors could impact reproducibility and standardization in clinical applications. Addressing these limitations through in vivo studies and optimized SVF isolation protocols will be essential for future translation. Therefore, further in vivo studies should be conducted to assess the histological and functional roles of the full-thickness artificial skin developed in this study, focusing on its efficacy in wound closure, vascularization, host integration, potential immunogenicity issues related to bovine collagen, and associated immune responses.

Despite these limitations, the tri-layered full-thickness artificial skin with a basement membrane for wound healing developed in this study offers unique advancements in mechanical stability, structural biomimicry, and biocompatibility for developing full-thickness artificial skin for wound healing. The scaffold’s ability to maintain mechanical properties post-sterilization and its resistance to enzymatic degradation particularly highlight its clinical viability. Future research should focus on validating these constructs in vivo, exploring their vascularization potential, and establishing standardized preparation protocols to facilitate clinical adoption.

## 5. Conclusions

This study presents a novel strategy for full-thickness artificial skin development, combining SVF cells and keratinocytes with an optimized collagen scaffold and a basement membrane with a collagen–elastin scaffold. By addressing the critical limitations of existing substitutes, including structural simplicity and extended fabrication times, this approach contributes to advancing regenerative medicine and developing functional skin substitutes for clinical applications.

## Data Availability

The data presented in this article are not publicly available because the patient included in this study provided consent solely for academic publication in journals to contribute to medical advancements.

## Abbreviations

The following abbreviations are used in this manuscript:

CCK-8	Cell Counting Kit-8
SEM	Scanning Electron Microscopy
SVF	Stromal Vascular Fraction
3D	Three-dimensional
